# The Book World of Medicine and Science

**Published:** 1903-07-25

**Authors:** 


### THE BOOK WORLD OF MEDICINE AND SCIENCE.

OPERATIVE SURGERY. By HERBERT W. ALLINGHAM, F.R.C.S. (London: Baillière, Tindall, and Cox. 1903. Pp. 367, with 215 illustrations. 7s. 6d. net.)

THE handy size and pleasing appearance of this book, together with the clearness of the type, combine to give the reader a good impression to commence with, but it must be admitted that on a further acquaintance with the contents the volume fails in some respects to fulfil our expectations. Although it is a pretty well recognised fact that the best clinical teacher is not always the most successful maker of books, yet one cannot but feel somewhat disappointed that Mr. Allingham has not done himself better justice. In the preface the author says that he hopes the work will be of service to surgeons wishing to hastily look up the leading features of an operation, and also to those who are performing operations upon the cadaver. For our own part we cannot altogether avoid the belief that he would be a hardy man who would trust to the rather meagre details to be found in these pages in order to refresh his memory before performing any but the mildest procedure, and it must be admitted that the book would be of greater value if more space were given to an account of the various pitfalls, dangers and complications which may be met with in the course of the different operations described. These are matters to which every surgeon is glad to have his memory recalled, and yet they either are not mentioned at all or else receive only the scantiest notice. The advice given is often vague and indecisive; for example, five different operations for amputation through the shoulder-joint are dealt with, all of them in the briefest possible manner, and yet not a word is said about the comparative advantages or drawbacks of the respective methods, and the inquirer is left to form his own conclusions entirely as to which is the best method for him to adopt. Nor is it always easy to be in accordance with the recommendations which are given. In dealing with the removal of the superior maxilla, Mr. Allingham tells us that "the best procedure is to start by performing tracheotomy and ligature of the external carotid; a sponge put in the pharynx prevents blood getting into the trachea, and ligaturing the external carotid makes the operation almost bloodless." Surely this advice must only be intended for the student who is operating upon the cadaver? As to the general style of the book it is unconvincing and in places illogical, added to which there is a noticeable want of accuracy in description; for instance, after recounting an operation under the heading of "Gritti's or Stokes' amputation"—as though the two were identical procedures—it is added that "the only difference between Gritti's and Stokes' operation is that, strictly speaking, Gritti saws through the condyles and Stokes saws through the lower end of the femur." The errata are numerous, thus Skey's operation is described in several places as Skeg's operation, while fig. 17 on p 33 would sorely tax the nerves of any student whose knowledge of the anatomy of the extensor tendons about the wrist-joint rests on the flimsy basis of a mnemonic, for no fewer than eight errata have crept into the illustration. No doubt the book will have a use among those for whom it seems to be principally intended, namely students who are performing operations on the dead body.

REFRACTION OF THE EYE AND ANOMALIES OF THE OCULAR MUSCLES. By KENNETH CAMPBELL. (Pp. 214. Illustrations 107. 5s.)

THE author may be said to have been successful in carrying out Professor Tait's principle, "True science is itself simple and should be explained in as simple and definite language as possible." There is no doubt that to the beginner the refraction of the eye presents many difficulties which are not cleared up by reference to text-books on the subject, but are gradually disclosed by practical experience. The work may be considered a short cut and is as practical as a work of the nature could possibly be; the descriptions are made in the simplest language and the style is terse. Two chapters are devoted to optics and their relation to ophthalmology. A useful diagram well illustrates the angles alpha and gamma. The following chapters are on accommodation and convergence, the ophthalmoscope and the normal fundus; the remaining ones are devoted to refraction and defects of the ocular muscles. The pages are interspersed with paragraphs in small type, which one must suppose have reference to relatively unimportant matter; the importance of the fact that ciliary spasm causes hypermetropia to simulate myopia, is so important for the beginner to realise fully, that it deserves italics rather than small type. The treatment of hypermetropia, astigmatism, and myopia is singularly clear, not only as regards the ordering and wearing of glasses, but in regard to reading, writing, the effect of various lights, and general health. There is no mention of the fact that cylinders may be worn in any frames other than spectacles. It is important to remember that many young women prefer to suffer rather than wear spectacles, whereas they might be persuaded to wear pince-nez: the only form of pince-nez which can carry cylinders is the so-called "novel nipper" with a spring on the top and which is non-folding. The important and difficult subject of asthenopia is dealt with in a lengthy chapter. The different tests for heterophoria are very lucidly described, and the uses and abuses of prisms are dealt with clearly. Concomitant squint and its treatment with a view to re-establishing binocular vision will be an aid to many who have found great difficulty in treating this condition. On the whole this is a very useful book to the student who wishes to master the refraction of the eye.

ELEMENTARY OPHTHALMIC OPTICS. By FREELAND FERGUS, M.D., F.R.S.E. (Blackie and Son. Pp. 106. Price 3s. 6d.)

THIS small volume is essentially one of physical and geometrical optics, and as such it sets forth the principles clearly. That the long-suffering medical student should, as is suggested in the preface, be expected to study this work may be good advice, but it is hardly likely to be followed, when we consider the mass of more necessary literature and the requirements of modern examinations. To the budding specialist in ophthalmology with a mathematical trend this and kindred works are very necessary for a clear conception of all the problems met with in ophthalmology.

